# 2′-Amino-1′-(4-chloro­phen­yl)-1,7′,7′-trimethyl-2,5′-dioxo-5′,6′,7′,8′-tetra­hydrospiro­[indoline-3,4′(1′*H*)-quinoline]-3′-carbonitrile dimethyl­formamide solvate dihydrate

**DOI:** 10.1107/S1600536809010551

**Published:** 2009-03-28

**Authors:** Jing Wang, Song-Lei Zhu

**Affiliations:** a Department of Chemistry, Xuzhou Medical College, Xuzhou 221002, People’s Republic of China

## Abstract

In the mol­ecule of the title compound, C_26_H_23_ClN_4_O_2_·C_3_H_7_NO·2H_2_O, the indole and dihydro­pyridine rings are planar and make a dihedral angle of 89.86 (7)°. The dihydro­pyridine ring forms a dihedral angle of 79.95 (7)° with the attached benzene ring. In the crystal structure, inter­molecular N—H⋯O and O—H⋯O hydrogen bonds link the mol­ecules. Intermolecular C—H⋯N and C—H⋯Cl interactions are also present.

## Related literature

For the indole nucleus, see: da Silva *et al.* (2001 ▶). For the anti­bacterial and fungicidal activities of indole compounds, see: Joshi & Chand (1982 ▶). For spiro­oxindole ring systems in alkaloids, see: Abdel-Rahman *et al.* (2004 ▶). For the preparation of heterocyclic compounds involving indole derivatives, see: Zhu *et al.* (2007 ▶).
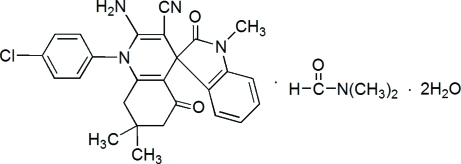


## Experimental

### 

#### Crystal data

C_26_H_23_ClN_4_O_2_·C_3_H_7_NO·2H_2_O
*M* *_r_* = 568.06
Triclinic, 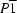

*a* = 9.237 (1) Å
*b* = 12.9553 (17) Å
*c* = 14.4554 (11) Å
α = 66.162 (11)°
β = 71.619 (12)°
γ = 84.595 (15)°
*V* = 1500.5 (3) Å^3^
*Z* = 2
Mo *K*α radiation
μ = 0.17 mm^−1^
*T* = 293 K
0.60 × 0.57 × 0.30 mm

#### Data collection

Rigaku Mercury diffractometer
Absorption correction: multi-scan (Jacobson, 1998 ▶) *T*_min_ = 0.760, *T*_max_ = 0.950
14714 measured reflections
5445 independent reflections
4310 reflections with *I* > 2σ(*I*)
*R*_int_ = 0.028

#### Refinement

*R*[*F* ^2^ > 2σ(*F* ^2^)] = 0.066
*wR*(*F* ^2^) = 0.155
*S* = 1.13
5445 reflections
379 parameters
4 restraints
H atoms treated by a mixture of independent and constrained refinement
Δρ_max_ = 0.21 e Å^−3^
Δρ_min_ = −0.31 e Å^−3^

### 

Data collection: *CrystalClear* (Rigaku/MSC, 2001 ▶); cell refinement: *CrystalClear*; data reduction: *CrystalStructure* (Rigaku/MSC, 2004 ▶); program(s) used to solve structure: *SHELXS97* (Sheldrick, 2008 ▶); program(s) used to refine structure: *SHELXL97* (Sheldrick, 2008 ▶); molecular graphics: *ORTEPII* (Johnson, 1976 ▶); software used to prepare material for publication: *SHELXL97*.

## Supplementary Material

Crystal structure: contains datablocks global, I. DOI: 10.1107/S1600536809010551/bq2126sup1.cif

Structure factors: contains datablocks I. DOI: 10.1107/S1600536809010551/bq2126Isup2.hkl

Additional supplementary materials:  crystallographic information; 3D view; checkCIF report

## Figures and Tables

**Table 1:** Hydrogen-bond geometry (Å, °)

*D*—H⋯*A*	*D*—H	H⋯*A*	*D*⋯*A*	*D*—H⋯*A*
C28—H28*B*⋯N3^i^	0.96	2.60	3.528 (5)	163
C15—H15⋯Cl1^ii^	0.93	2.74	3.647 (3)	167
N4—H4*D*⋯O4^iii^	0.86	2.23	2.934 (3)	139
N4—H4*C*⋯O3^iv^	0.86	2.24	3.071 (3)	162
O5—H5*B*⋯O3	0.82 (4)	2.03 (4)	2.830 (4)	163 (4)
O5—H5*A*⋯O1	0.83 (3)	2.017 (16)	2.816 (3)	164 (4)
O4—H4*B*⋯O2^v^	0.82 (3)	2.17 (3)	2.974 (3)	167 (4)
O4—H4*A*⋯O5^vi^	0.82 (4)	1.99 (4)	2.800 (4)	170 (4)

Symmetry codes: (i) 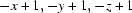; (ii) 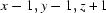; (iii) 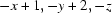; (iv) 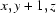; (v) 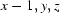; (vi) 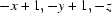.

**Fig. 1.**
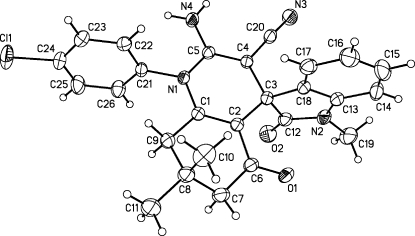
The molecular structure of the title molecule, with the atom-numbering scheme. Displacement ellipsoids are drawn at the 30% probability level. The solvent DMF and the two water molecules are not shown for clarity.

**Fig. 2.**
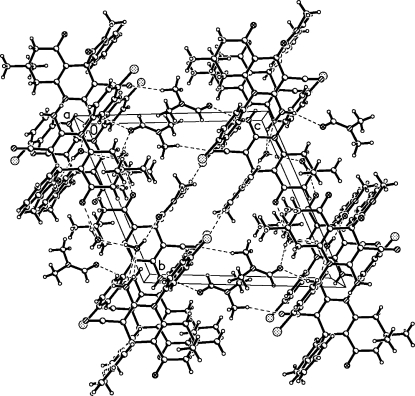
A packing diagram of (I). Hydrogen bonds are shown as dashed lines.

## supplementary crystallographic information

### Comment

The indole nucleus is the well known heterocyclic compound (da Silva *et al.*, 2001). Compounds carrying the indole moiety exhibit antibacterial and fungicidal activities (Joshi & Chand, 1982). Spirooxindole ring systems are found in a number of alkaloids like horsifiline, spirotryprostatin and elacomine (Abdel-Rahman *et al.*, 2004). As a part of our program devoted to the preparation of heterocyclic compounds involving indole derivatives (Zhu *et al.*,  2007), we have synthesized a series of spirooxindoles *via* reactions of substituted isatins together with malononitrile and enaminones. We report herein the crystal structure of the title compound, (I).

In the molecule of (I), (Fig. 1), the indole ring A (C3/C12/N2/C13-C18) and the dihydropyridine ring B (N1/C1-C5), are planar. The dihedral angle between them is 89.86 (7)°, and the benzene ring C (C21-C26) is oriented at a dihedral angle of 79.95 (7)° with the attached ring B. Ring D (C1/C2/C6-C9) adopts twisted conformation, with C8 deviating the C1/C2/C6/C7 plan by 0.636 (3)Å.

In the crystal structure, intermolecular N-H···O and O-H···O, hydrogen bonds link the molecules (Fig. 2), in which they may be effective in the stabilization of the structure.

### Experimental

Compound (I) was prepared by one-pot reaction of 1-methylisatin (2 mmol), malononitrile (2 mmol) and 3-(4-chlorophenylamino) -5,5-dimethylcyclohex-2-enone (2 mmol) in ethanol. After stirring at 343 K for 5 h, the reaction mixture was cooled and washed with small amount of ethanol. The crude product was filtered and single crystals of the title compound were obtained from DMF and water mixture solution by slow evaporation at room temperature (yield; 80%, m.p. > 573 K). Spectroscopic analysis: IR (KBr, n, cm^-1^): 3463, 3312, 2190, 1716, 1650, 1568, 1491, 1364, 1090, 1018, 915, 753. ^1^H NMR (400 MHz, DMSO-d_6_): 7.65 (d, J = 8.4 Hz, 2H, Ar-H), 7.51-7.54 (m, 2H, ArH), 7.23 (t, J = 8.0 Hz, 2H, ArH), 6.93-6.70 (m, 2H, ArH), 5.55 (s, 2H, NH_2_), 3.13 (s, 3H, NCH_3_), 2.05-2.17 (m, 2H, CH_2_), 1.81-1.93 (m, 2H, CH_2_), 0.89 (s, 3H, CH_3_), 0.81 (s, 3H, CH_3_).

### Refinement

H atoms were positioned geometrically, with N-H=0.86Å (for NH) and C-H=0.93Å and 0.97Å for aromatic and methyl H and constrained to ride on their parent atoms with U_iso_(H)=xU_eq_(C,N), where x=1.5 for methyl H and x=1.2 for all other H atoms.

### Crystal data

**Table:**

C_26_H_23_ClN_4_O_2_·C_3_H_7_NO·2(H_2_O)	*Z* = 2
*M**_r_* = 568.06	*F*(000) = 600
Triclinic, *P*1	*D*_x_ = 1.257 Mg m^−^^3^
Hall symbol: -P 1	Melting point > 573 K
*a* = 9.237 (1) Å	Mo *K*α radiation, λ = 0.71070 Å
*b* = 12.9553 (17) Å	Cell parameters from 5610 reflections
*c* = 14.4554 (11) Å	θ = 3.0–25.3°
α = 66.162 (11)°	µ = 0.17 mm^−^^1^
β = 71.619 (12)°	*T* = 293 K
γ = 84.595 (15)°	Block, colorless
*V* = 1500.5 (3) Å^3^	0.60 × 0.57 × 0.30 mm

### Data collection

**Table:**

Rigaku Mercury diffractometer	5445 independent reflections
Radiation source: fine-focus sealed tube	4310 reflections with *I* > 2σ(*I*)
graphite	*R*_int_ = 0.028
Detector resolution: 7.31 pixels mm^-1^	θ_max_ = 25.4°, θ_min_ = 3.0°
ω scans	*h* = −10→11
Absorption correction: multi-scan (Jacobson, 1998)	*k* = −15→15
*T*_min_ = 0.760, *T*_max_ = 0.950	*l* = −16→17
14714 measured reflections	

### Refinement

**Table:**

Refinement on *F*^2^	Primary atom site location: structure-invariant direct methods
Least-squares matrix: full	Secondary atom site location: difference Fourier map
*R*[*F*^2^ > 2σ(*F*^2^)] = 0.066	Hydrogen site location: inferred from neighbouring sites
*wR*(*F*^2^) = 0.155	H atoms treated by a mixture of independent and constrained refinement
*S* = 1.13	*w* = 1/[σ^2^(*F*_o_^2^) + (0.0606*P*)^2^ + 0.5335*P*] where *P* = (*F*_o_^2^ + 2*F*_c_^2^)/3
5445 reflections	(Δ/σ)_max_ = 0.001
379 parameters	Δρ_max_ = 0.21 e Å^−^^3^
4 restraints	Δρ_min_ = −0.31 e Å^−^^3^

### Special details

**Table:**

Geometry. All esds (except the esd in the dihedral angle between two l.s. planes) are estimated using the full covariance matrix. The cell esds are taken into account individually in the estimation of esds in distances, angles and torsion angles; correlations between esds in cell parameters are only used when they are defined by crystal symmetry. An approximate (isotropic) treatment of cell esds is used for estimating esds involving l.s. planes.
Refinement. Refinement of F^2^ against ALL reflections. The weighted R-factor wR and goodness of fit S are based on F^2^, conventional R-factors R are based on F, with F set to zero for negative F^2^. The threshold expression of F^2^ > 2sigma(F^2^) is used only for calculating R-factors(gt) etc. and is not relevant to the choice of reflections for refinement. R-factors based on F^2^ are statistically about twice as large as those based on F, and R- factors based on ALL data will be even larger.

### Fractional atomic coordinates and isotropic or equivalent isotropic displacement parameters (Å^2^)

**Table:**

	*x*	*y*	*z*	*U*_iso_*/*U*_eq_	
Cl1	0.98120 (12)	1.26623 (8)	−0.42483 (7)	0.1045 (4)	
O1	0.7477 (2)	0.47841 (13)	0.12855 (14)	0.0562 (5)	
O2	0.9204 (2)	0.60921 (17)	0.22240 (15)	0.0609 (5)	
O3	0.5785 (4)	0.07513 (19)	0.2612 (2)	0.1004 (9)	
O4	0.1363 (4)	0.7917 (2)	0.0471 (2)	0.0956 (8)	
H4A	0.148 (5)	0.772 (4)	−0.002 (2)	0.115*	
H4B	0.076 (4)	0.747 (3)	0.101 (2)	0.115*	
O5	0.7925 (3)	0.2586 (2)	0.1329 (2)	0.0918 (8)	
H5A	0.781 (5)	0.317 (2)	0.144 (3)	0.110*	
H5B	0.721 (3)	0.214 (3)	0.176 (3)	0.110*	
N1	0.7639 (2)	0.87742 (14)	−0.01571 (14)	0.0364 (4)	
N2	0.6913 (3)	0.51570 (16)	0.33166 (15)	0.0490 (5)	
N3	0.5786 (3)	0.79554 (19)	0.35627 (18)	0.0595 (6)	
N4	0.7018 (2)	0.98819 (15)	0.08330 (15)	0.0436 (5)	
H4C	0.6681	0.9967	0.1424	0.052*	
H4D	0.7271	1.0464	0.0242	0.052*	
N5	0.3930 (5)	0.0797 (3)	0.4056 (3)	0.1189 (14)	
C1	0.7780 (2)	0.77324 (18)	−0.02435 (17)	0.0351 (5)	
C2	0.7457 (2)	0.67493 (18)	0.06225 (17)	0.0357 (5)	
C3	0.6836 (2)	0.66875 (18)	0.17425 (17)	0.0361 (5)	
C4	0.6775 (2)	0.78666 (18)	0.17317 (17)	0.0348 (5)	
C5	0.7150 (2)	0.88302 (17)	0.08337 (17)	0.0336 (5)	
C6	0.7733 (3)	0.5680 (2)	0.04972 (19)	0.0422 (6)	
C7	0.8399 (3)	0.5698 (2)	−0.0597 (2)	0.0521 (7)	
H7A	0.8078	0.5007	−0.0600	0.063*	
H7B	0.9504	0.5711	−0.0777	0.063*	
C8	0.7935 (3)	0.6698 (2)	−0.1438 (2)	0.0499 (6)	
C9	0.8308 (3)	0.7769 (2)	−0.13491 (18)	0.0457 (6)	
H9A	0.9405	0.7912	−0.1630	0.055*	
H9B	0.7845	0.8399	−0.1790	0.055*	
C10	0.6231 (4)	0.6592 (3)	−0.1279 (3)	0.0723 (9)	
H10A	0.6032	0.5937	−0.1380	0.108*	
H10B	0.5932	0.7254	−0.1785	0.108*	
H10C	0.5659	0.6519	−0.0573	0.108*	
C11	0.8872 (4)	0.6759 (3)	−0.2544 (2)	0.0767 (10)	
H11A	0.9938	0.6831	−0.2636	0.115*	
H11B	0.8585	0.7402	−0.3074	0.115*	
H11C	0.8678	0.6083	−0.2614	0.115*	
C12	0.7833 (3)	0.5956 (2)	0.24294 (19)	0.0439 (6)	
C13	0.5405 (3)	0.52001 (19)	0.32746 (19)	0.0457 (6)	
C14	0.4138 (4)	0.4527 (2)	0.4012 (2)	0.0673 (9)	
H14	0.4199	0.3940	0.4636	0.081*	
C15	0.2771 (4)	0.4775 (3)	0.3771 (3)	0.0768 (10)	
H15	0.1900	0.4339	0.4250	0.092*	
C16	0.2652 (3)	0.5628 (3)	0.2865 (3)	0.0700 (9)	
H16	0.1714	0.5767	0.2735	0.084*	
C17	0.3932 (3)	0.6290 (2)	0.2137 (2)	0.0506 (6)	
H17	0.3866	0.6874	0.1512	0.061*	
C18	0.5295 (3)	0.60722 (18)	0.23519 (18)	0.0387 (5)	
C19	0.7487 (4)	0.4264 (3)	0.4093 (2)	0.0743 (9)	
H19A	0.7541	0.3587	0.3967	0.111*	
H19B	0.6814	0.4131	0.4792	0.111*	
H19C	0.8487	0.4482	0.4035	0.111*	
C20	0.6241 (3)	0.79382 (19)	0.27326 (19)	0.0413 (6)	
C21	0.8148 (2)	0.97855 (18)	−0.11100 (17)	0.0350 (5)	
C22	0.7118 (3)	1.04436 (19)	−0.15805 (19)	0.0432 (6)	
H22	0.6077	1.0277	−0.1254	0.052*	
C23	0.7629 (3)	1.1353 (2)	−0.2540 (2)	0.0523 (7)	
H23	0.6939	1.1808	−0.2863	0.063*	
C24	0.9163 (3)	1.1575 (2)	−0.3009 (2)	0.0554 (7)	
C25	1.0204 (3)	1.0947 (2)	−0.2537 (2)	0.0598 (7)	
H25	1.1243	1.1127	−0.2860	0.072*	
C26	0.9691 (3)	1.0041 (2)	−0.1572 (2)	0.0492 (6)	
H26	1.0382	0.9607	−0.1237	0.059*	
C27	0.4592 (6)	0.0402 (3)	0.3331 (3)	0.0904 (12)	
H27	0.4101	−0.0214	0.3366	0.108*	
C28	0.4561 (9)	0.1747 (5)	0.4057 (5)	0.187 (3)	
H28A	0.5649	0.1780	0.3746	0.280*	
H28B	0.4322	0.1686	0.4774	0.280*	
H28C	0.4141	0.2420	0.3652	0.280*	
C29	0.2537 (8)	0.0312 (7)	0.4884 (5)	0.205 (4)	
H29A	0.2198	−0.0318	0.4806	0.307*	
H29B	0.1771	0.0869	0.4839	0.307*	
H29C	0.2710	0.0060	0.5562	0.307*	

### Atomic displacement parameters (Å^2^)

**Table:**

	*U*^11^	*U*^22^	*U*^33^	*U*^12^	*U*^13^	*U*^23^
Cl1	0.1137 (8)	0.0718 (6)	0.0595 (5)	−0.0027 (5)	0.0020 (5)	0.0216 (4)
O1	0.0800 (13)	0.0305 (9)	0.0487 (11)	0.0064 (8)	−0.0133 (9)	−0.0120 (8)
O2	0.0489 (11)	0.0686 (13)	0.0630 (12)	0.0095 (9)	−0.0250 (9)	−0.0194 (10)
O3	0.158 (3)	0.0573 (14)	0.0778 (18)	−0.0061 (15)	−0.0191 (18)	−0.0296 (13)
O4	0.133 (2)	0.0648 (15)	0.0736 (18)	−0.0345 (14)	−0.0062 (16)	−0.0228 (13)
O5	0.0887 (18)	0.0642 (16)	0.126 (2)	0.0034 (13)	−0.0146 (15)	−0.0546 (16)
N1	0.0479 (11)	0.0275 (10)	0.0295 (10)	−0.0011 (8)	−0.0091 (8)	−0.0086 (8)
N2	0.0682 (14)	0.0371 (11)	0.0335 (11)	0.0104 (10)	−0.0172 (10)	−0.0063 (9)
N3	0.0835 (17)	0.0514 (14)	0.0413 (13)	0.0010 (11)	−0.0124 (12)	−0.0209 (11)
N4	0.0590 (13)	0.0328 (10)	0.0358 (11)	−0.0035 (9)	−0.0092 (9)	−0.0131 (9)
N5	0.197 (4)	0.101 (3)	0.075 (2)	0.080 (3)	−0.058 (3)	−0.055 (2)
C1	0.0369 (12)	0.0329 (12)	0.0343 (12)	0.0008 (9)	−0.0100 (10)	−0.0128 (10)
C2	0.0401 (12)	0.0329 (12)	0.0333 (12)	0.0023 (9)	−0.0107 (10)	−0.0127 (10)
C3	0.0401 (12)	0.0314 (12)	0.0331 (12)	−0.0001 (9)	−0.0092 (10)	−0.0104 (10)
C4	0.0401 (12)	0.0315 (12)	0.0311 (12)	−0.0018 (9)	−0.0082 (9)	−0.0121 (10)
C5	0.0344 (12)	0.0301 (11)	0.0365 (12)	−0.0005 (9)	−0.0108 (9)	−0.0129 (10)
C6	0.0507 (14)	0.0343 (13)	0.0415 (14)	0.0059 (10)	−0.0147 (11)	−0.0153 (11)
C7	0.0681 (17)	0.0425 (14)	0.0511 (16)	0.0109 (12)	−0.0194 (13)	−0.0248 (12)
C8	0.0699 (18)	0.0439 (14)	0.0421 (14)	0.0063 (12)	−0.0200 (13)	−0.0217 (12)
C9	0.0588 (15)	0.0411 (14)	0.0347 (13)	0.0019 (11)	−0.0113 (11)	−0.0147 (11)
C10	0.081 (2)	0.070 (2)	0.080 (2)	0.0025 (16)	−0.0420 (18)	−0.0305 (17)
C11	0.125 (3)	0.0619 (19)	0.0506 (18)	0.0082 (18)	−0.0236 (18)	−0.0325 (16)
C12	0.0545 (16)	0.0385 (13)	0.0375 (13)	0.0091 (11)	−0.0159 (11)	−0.0141 (11)
C13	0.0562 (16)	0.0327 (13)	0.0417 (14)	−0.0007 (11)	−0.0042 (12)	−0.0159 (11)
C14	0.094 (2)	0.0401 (15)	0.0464 (17)	−0.0152 (15)	0.0039 (16)	−0.0107 (13)
C15	0.062 (2)	0.069 (2)	0.085 (2)	−0.0284 (17)	0.0098 (18)	−0.034 (2)
C16	0.0542 (18)	0.068 (2)	0.092 (3)	−0.0113 (15)	−0.0084 (16)	−0.043 (2)
C17	0.0484 (15)	0.0431 (14)	0.0641 (17)	0.0001 (11)	−0.0155 (13)	−0.0258 (13)
C18	0.0437 (13)	0.0307 (12)	0.0382 (13)	−0.0026 (10)	−0.0060 (10)	−0.0143 (10)
C19	0.109 (3)	0.0574 (18)	0.0457 (17)	0.0280 (17)	−0.0305 (17)	−0.0095 (14)
C20	0.0504 (14)	0.0316 (12)	0.0392 (14)	−0.0024 (10)	−0.0120 (11)	−0.0116 (10)
C21	0.0405 (13)	0.0309 (11)	0.0293 (11)	−0.0043 (9)	−0.0080 (10)	−0.0084 (9)
C22	0.0387 (13)	0.0386 (13)	0.0454 (14)	0.0048 (10)	−0.0097 (11)	−0.0130 (11)
C23	0.0582 (17)	0.0428 (14)	0.0461 (15)	0.0105 (12)	−0.0186 (13)	−0.0079 (12)
C24	0.0668 (18)	0.0381 (14)	0.0413 (15)	−0.0031 (12)	−0.0086 (13)	−0.0008 (11)
C25	0.0456 (15)	0.0581 (17)	0.0542 (17)	−0.0143 (13)	−0.0035 (13)	−0.0062 (14)
C26	0.0444 (14)	0.0496 (15)	0.0467 (15)	−0.0026 (11)	−0.0168 (12)	−0.0091 (12)
C27	0.144 (4)	0.067 (2)	0.070 (2)	0.030 (2)	−0.039 (2)	−0.038 (2)
C28	0.386 (11)	0.122 (4)	0.147 (5)	0.106 (6)	−0.168 (6)	−0.102 (4)
C29	0.208 (7)	0.245 (8)	0.109 (4)	0.123 (6)	−0.015 (5)	−0.068 (5)

### Geometric parameters (Å, °)

**Table:**

Cl1—C24	1.730 (3)	C9—H9B	0.9700
O1—C6	1.230 (3)	C10—H10A	0.9600
O2—C12	1.219 (3)	C10—H10B	0.9600
O3—C27	1.221 (5)	C10—H10C	0.9600
O4—H4A	0.82 (4)	C11—H11A	0.9600
O4—H4B	0.82 (3)	C11—H11B	0.9600
O5—H5A	0.83 (3)	C11—H11C	0.9600
O5—H5B	0.82 (4)	C13—C18	1.383 (3)
N1—C5	1.389 (3)	C13—C14	1.386 (4)
N1—C1	1.395 (3)	C14—C15	1.389 (5)
N1—C21	1.444 (3)	C14—H14	0.9300
N2—C12	1.357 (3)	C15—C16	1.358 (5)
N2—C13	1.408 (3)	C15—H15	0.9300
N2—C19	1.445 (3)	C16—C17	1.385 (4)
N3—C20	1.148 (3)	C16—H16	0.9300
N4—C5	1.356 (3)	C17—C18	1.368 (3)
N4—H4C	0.8600	C17—H17	0.9300
N4—H4D	0.8600	C19—H19A	0.9600
N5—C27	1.306 (4)	C19—H19B	0.9600
N5—C28	1.410 (7)	C19—H19C	0.9600
N5—C29	1.434 (7)	C21—C22	1.372 (3)
C1—C2	1.351 (3)	C21—C26	1.377 (3)
C1—C9	1.499 (3)	C22—C23	1.381 (3)
C2—C6	1.458 (3)	C22—H22	0.9300
C2—C3	1.508 (3)	C23—C24	1.365 (4)
C3—C18	1.514 (3)	C23—H23	0.9300
C3—C4	1.517 (3)	C24—C25	1.367 (4)
C3—C12	1.544 (3)	C25—C26	1.384 (4)
C4—C5	1.361 (3)	C25—H25	0.9300
C4—C20	1.412 (3)	C26—H26	0.9300
C6—C7	1.497 (3)	C27—H27	0.9300
C7—C8	1.514 (4)	C28—H28A	0.9600
C7—H7A	0.9700	C28—H28B	0.9600
C7—H7B	0.9700	C28—H28C	0.9600
C8—C9	1.524 (3)	C29—H29A	0.9600
C8—C10	1.528 (4)	C29—H29B	0.9600
C8—C11	1.533 (4)	C29—H29C	0.9600
C9—H9A	0.9700		
			
H4A—O4—H4B	110 (4)	H11A—C11—H11C	109.5
H5A—O5—H5B	108 (4)	H11B—C11—H11C	109.5
C5—N1—C1	120.56 (17)	O2—C12—N2	125.9 (2)
C5—N1—C21	120.44 (17)	O2—C12—C3	125.8 (2)
C1—N1—C21	118.74 (18)	N2—C12—C3	108.3 (2)
C12—N2—C13	111.0 (2)	C18—C13—C14	121.1 (3)
C12—N2—C19	123.1 (2)	C18—C13—N2	110.0 (2)
C13—N2—C19	125.0 (2)	C14—C13—N2	128.9 (3)
C5—N4—H4C	120.0	C13—C14—C15	116.5 (3)
C5—N4—H4D	120.0	C13—C14—H14	121.7
H4C—N4—H4D	120.0	C15—C14—H14	121.7
C27—N5—C28	120.9 (5)	C16—C15—C14	122.9 (3)
C27—N5—C29	123.0 (5)	C16—C15—H15	118.6
C28—N5—C29	116.1 (5)	C14—C15—H15	118.6
C2—C1—N1	121.7 (2)	C15—C16—C17	119.8 (3)
C2—C1—C9	122.1 (2)	C15—C16—H16	120.1
N1—C1—C9	116.17 (19)	C17—C16—H16	120.1
C1—C2—C6	119.9 (2)	C18—C17—C16	118.9 (3)
C1—C2—C3	123.27 (19)	C18—C17—H17	120.6
C6—C2—C3	116.87 (19)	C16—C17—H17	120.6
C2—C3—C18	113.90 (18)	C17—C18—C13	120.9 (2)
C2—C3—C4	109.84 (17)	C17—C18—C3	130.3 (2)
C18—C3—C4	110.71 (17)	C13—C18—C3	108.7 (2)
C2—C3—C12	111.04 (18)	N2—C19—H19A	109.5
C18—C3—C12	101.58 (18)	N2—C19—H19B	109.5
C4—C3—C12	109.47 (18)	H19A—C19—H19B	109.5
C5—C4—C20	119.5 (2)	N2—C19—H19C	109.5
C5—C4—C3	124.29 (19)	H19A—C19—H19C	109.5
C20—C4—C3	116.16 (18)	H19B—C19—H19C	109.5
N4—C5—C4	123.7 (2)	N3—C20—C4	177.4 (2)
N4—C5—N1	116.05 (19)	C22—C21—C26	120.6 (2)
C4—C5—N1	120.16 (19)	C22—C21—N1	120.5 (2)
O1—C6—C2	120.0 (2)	C26—C21—N1	118.8 (2)
O1—C6—C7	121.1 (2)	C21—C22—C23	119.9 (2)
C2—C6—C7	118.9 (2)	C21—C22—H22	120.1
C6—C7—C8	113.4 (2)	C23—C22—H22	120.1
C6—C7—H7A	108.9	C24—C23—C22	119.0 (2)
C8—C7—H7A	108.9	C24—C23—H23	120.5
C6—C7—H7B	108.9	C22—C23—H23	120.5
C8—C7—H7B	108.9	C23—C24—C25	121.9 (2)
H7A—C7—H7B	107.7	C23—C24—Cl1	119.1 (2)
C7—C8—C9	108.1 (2)	C25—C24—Cl1	119.0 (2)
C7—C8—C10	110.0 (2)	C24—C25—C26	119.1 (2)
C9—C8—C10	110.9 (2)	C24—C25—H25	120.5
C7—C8—C11	109.8 (2)	C26—C25—H25	120.5
C9—C8—C11	107.8 (2)	C21—C26—C25	119.5 (2)
C10—C8—C11	110.2 (2)	C21—C26—H26	120.3
C1—C9—C8	114.7 (2)	C25—C26—H26	120.3
C1—C9—H9A	108.6	O3—C27—N5	127.2 (4)
C8—C9—H9A	108.6	O3—C27—H27	116.4
C1—C9—H9B	108.6	N5—C27—H27	116.4
C8—C9—H9B	108.6	N5—C28—H28A	109.5
H9A—C9—H9B	107.6	N5—C28—H28B	109.5
C8—C10—H10A	109.5	H28A—C28—H28B	109.5
C8—C10—H10B	109.5	N5—C28—H28C	109.5
H10A—C10—H10B	109.5	H28A—C28—H28C	109.5
C8—C10—H10C	109.5	H28B—C28—H28C	109.5
H10A—C10—H10C	109.5	N5—C29—H29A	109.5
H10B—C10—H10C	109.5	N5—C29—H29B	109.5
C8—C11—H11A	109.5	H29A—C29—H29B	109.5
C8—C11—H11B	109.5	N5—C29—H29C	109.5
H11A—C11—H11B	109.5	H29A—C29—H29C	109.5
C8—C11—H11C	109.5	H29B—C29—H29C	109.5
			
C5—N1—C1—C2	−0.3 (3)	C2—C3—C12—O2	54.8 (3)
C21—N1—C1—C2	173.8 (2)	C18—C3—C12—O2	176.3 (2)
C5—N1—C1—C9	179.7 (2)	C4—C3—C12—O2	−66.6 (3)
C21—N1—C1—C9	−6.2 (3)	C2—C3—C12—N2	−127.1 (2)
N1—C1—C2—C6	−175.3 (2)	C18—C3—C12—N2	−5.6 (2)
C9—C1—C2—C6	4.8 (3)	C4—C3—C12—N2	111.5 (2)
N1—C1—C2—C3	3.7 (3)	C12—N2—C13—C18	−1.9 (3)
C9—C1—C2—C3	−176.2 (2)	C19—N2—C13—C18	−171.3 (2)
C1—C2—C3—C18	119.9 (2)	C12—N2—C13—C14	179.3 (2)
C6—C2—C3—C18	−61.1 (3)	C19—N2—C13—C14	10.0 (4)
C1—C2—C3—C4	−4.9 (3)	C18—C13—C14—C15	0.2 (4)
C6—C2—C3—C4	174.08 (19)	N2—C13—C14—C15	178.8 (3)
C1—C2—C3—C12	−126.2 (2)	C13—C14—C15—C16	0.0 (5)
C6—C2—C3—C12	52.9 (3)	C14—C15—C16—C17	0.2 (5)
C2—C3—C4—C5	3.4 (3)	C15—C16—C17—C18	−0.4 (4)
C18—C3—C4—C5	−123.3 (2)	C16—C17—C18—C13	0.6 (4)
C12—C3—C4—C5	125.6 (2)	C16—C17—C18—C3	−176.1 (2)
C2—C3—C4—C20	−178.80 (19)	C14—C13—C18—C17	−0.5 (4)
C18—C3—C4—C20	54.6 (3)	N2—C13—C18—C17	−179.3 (2)
C12—C3—C4—C20	−56.6 (3)	C14—C13—C18—C3	176.9 (2)
C20—C4—C5—N4	−1.2 (3)	N2—C13—C18—C3	−2.0 (3)
C3—C4—C5—N4	176.5 (2)	C2—C3—C18—C17	−59.0 (3)
C20—C4—C5—N1	−178.3 (2)	C4—C3—C18—C17	65.4 (3)
C3—C4—C5—N1	−0.6 (3)	C12—C3—C18—C17	−178.5 (2)
C1—N1—C5—N4	−178.59 (19)	C2—C3—C18—C13	123.9 (2)
C21—N1—C5—N4	7.4 (3)	C4—C3—C18—C13	−111.7 (2)
C1—N1—C5—C4	−1.3 (3)	C12—C3—C18—C13	4.5 (2)
C21—N1—C5—C4	−175.3 (2)	C5—C4—C20—N3	163 (6)
C1—C2—C6—O1	178.2 (2)	C3—C4—C20—N3	−15 (6)
C3—C2—C6—O1	−0.9 (3)	C5—N1—C21—C22	−85.8 (3)
C1—C2—C6—C7	1.1 (3)	C1—N1—C21—C22	100.1 (3)
C3—C2—C6—C7	−178.0 (2)	C5—N1—C21—C26	98.0 (3)
O1—C6—C7—C8	151.3 (2)	C1—N1—C21—C26	−76.1 (3)
C2—C6—C7—C8	−31.7 (3)	C26—C21—C22—C23	1.8 (4)
C6—C7—C8—C9	53.2 (3)	N1—C21—C22—C23	−174.4 (2)
C6—C7—C8—C10	−68.0 (3)	C21—C22—C23—C24	0.5 (4)
C6—C7—C8—C11	170.5 (2)	C22—C23—C24—C25	−2.4 (4)
C2—C1—C9—C8	20.2 (3)	C22—C23—C24—Cl1	176.2 (2)
N1—C1—C9—C8	−159.8 (2)	C23—C24—C25—C26	1.9 (4)
C7—C8—C9—C1	−47.8 (3)	Cl1—C24—C25—C26	−176.6 (2)
C10—C8—C9—C1	72.9 (3)	C22—C21—C26—C25	−2.2 (4)
C11—C8—C9—C1	−166.4 (2)	N1—C21—C26—C25	174.0 (2)
C13—N2—C12—O2	−177.0 (2)	C24—C25—C26—C21	0.4 (4)
C19—N2—C12—O2	−7.5 (4)	C28—N5—C27—O3	1.6 (7)
C13—N2—C12—C3	4.9 (3)	C29—N5—C27—O3	−178.8 (5)
C19—N2—C12—C3	174.4 (2)		

### Hydrogen-bond geometry (Å, °)

**Table:**

*D*—H···*A*	*D*—H	H···*A*	*D*···*A*	*D*—H···*A*
C28—H28B···N3^i^	0.96	2.60	3.528 (5)	163
C15—H15···Cl1^ii^	0.93	2.74	3.647 (3)	167
N4—H4D···O4^iii^	0.86	2.23	2.934 (3)	139
N4—H4C···O3^iv^	0.86	2.24	3.071 (3)	162
O5—H5B···O3	0.82 (4)	2.03 (4)	2.830 (4)	163 (4)
O5—H5A···O1	0.83 (3)	2.02 (2)	2.816 (3)	164 (4)
O4—H4B···O2^v^	0.82 (3)	2.17 (3)	2.974 (3)	167 (4)
O4—H4A···O5^vi^	0.82 (4)	1.99 (4)	2.800 (4)	170 (4)

Symmetry codes: (i) −*x*+1, −*y*+1, −*z*+1; (ii) *x*−1, *y*−1, *z*+1; (iii) −*x*+1, −*y*+2, −*z*; (iv) *x*, *y*+1, *z*; (v) *x*−1, *y*, *z*; (vi) −*x*+1, −*y*+1, −*z*.
